# Spin‐Manipulated Photonic Skyrmion‐Pair for Pico‐Metric Displacement Sensing

**DOI:** 10.1002/advs.202205249

**Published:** 2023-02-25

**Authors:** Aiping Yang, Xinrui Lei, Peng Shi, Fanfei Meng, Min Lin, Luping Du, Xiaocong Yuan

**Affiliations:** ^1^ Nanophotonics Research Centre Institute of Microscale Optoelectronics & State Key Laboratory of Radio Frequency Heterogeneous Integration Shenzhen University Shenzhen 518060 P. R. China

**Keywords:** photonic skyrmions, pico‐metric displacement sensing, spin angular momentum, surface plasmon polaritons

## Abstract

Photonic spin skyrmions with deep‐subwavelength features have aroused considerable interest in recent years. However, the manipulation of spin structure in the skyrmions in a desired manner is still a challenge, while this is crucial for developing the skyrmion‐based applications. Here, an approach of optical spin manipulation by utilizing the spin‐momentum equation is proposed to investigate the spin texture in a photonic skyrmion‐pair. With the benefit of the proposed approach, a unique spin texture with spin angular momentum varying linearly along the line connecting the two skyrmion centers is theoretically designed and experimentally verified. The optimized spin texture is then applied in a displacement‐sensing system, which is capable of attaining pico‐metric sensitivity. Compared with the conventional polarization and phase schemes, the spin‐based manipulation mechanism provides a new pathway for optical modulation, which is of great value in nanophotonics from both fundamental and application.

## Graphical abstract

A robust, Angstrom‐scaled displacement‐sensing technique is proposed based on the structured spin texture in a photonic skyrmion‐pair, where the interaction between the skyrmions induces a linear change in spin texture within its central region over distances of hundred‐nanometers.

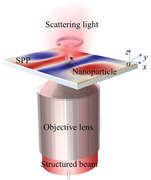

## 1 Introduction

Magnetic skyrmions as topological nontrivial spin textures of electrons have aroused tremendous interest in the past decades,^[^ ^1^, ^2^, ^3^, ^4^, ^5^ ^]^ which have shown great potential for applications in spintronics owing to their stability by topological protection and low driven current.^[^ ^6^, ^7^, ^8^ ^]^ Recently, the photonic counterparts of magnetic skyrmions, namely the photonic skyrmions, were discovered in confined electromagnetic fields either in the form of spin vector (photonic spin‐skyrmions)^[^ ^9^, ^10^, ^11^, ^12^, ^13^, ^14^, ^15^, ^16^, ^17^, ^18^ ^]^ or field vector (photonic field‐skyrmions).^[^ ^19^, ^20^, ^21^, ^22^, ^23^ ^]^ Photonic spin skyrmions manifest the topological properties in real space through the spin‐momentum locking in evanescent fields, and the deep‐subwavelength features in local spin distributions make them excellent for applications in optical sensing and metrology.^[^ ^9^, ^12^ ^]^

While possessing peculiar spin texture, manipulation of photonic skyrmions and their interactions is essential to modulate the spin structure in a desired manner. To this end, the generalized spin‐momentum relation between the spin angular momentum (SAM) and Poynting vector was established,^[^ ^24^ ^]^ where the SAMs are obtainable directly from a scalar wave function without analyzing the complex vector and phase information of the field, facilitating the construction of spin texture in a complex multiple skyrmion configuration. Based on this, many novel skyrmionic structures were generated, such as the formation of spin merons with the interaction between multiple skyrmions,^[^ ^10^, ^11^ ^]^ and metrology‐related applications were proposed by the superposed skyrmion states.^[^ ^12^ ^]^

Here by utilizing the spin‐momentum relationship, we demonstrated a spin manipulation approach to design a unique spin texture in a photonic skyrmion‐pair, which contains a distinct feature for applications. A spin texture with SAM varying linearly along the line connecting the centers of the two skyrmions was theoretically designed. By adjusting the spacing distance between the two photonic skyrmions, this SAM linear region could be optimized to enable an optical displacement sensing system with ultrahigh sensitivity. To verify this in an experiment, the spin structure in a skyrmion‐pair was excited by a specially designed vector beam and measured by our home‐built near‐field imaging system. This spin texture was then employed for displacement sensing, which is able to sustain pico‐metric sensitivity over distances of at least a hundred‐nanometers. This spin‐based manipulation provides a fresh perspective to study the coupling process between multiple skyrmions and enables an ultrahigh precision optical displacement sensing method. Compared with the conventional interferometric methods^[^ ^25^, ^26^ ^]^ and the approaches using electromagnetic field singularities,^[^ ^27^, ^28^, ^29^, ^30^ ^]^ this robust spin‐based displacement sensing avoids interferometric observations and weak signal measurements near singularities. These advantages make it have potential applications in far‐field imaging, localized nanoscopy, and quantum measurements.

## 2 Results

### 2.1 Analyzing of Spin Texture in the Skyrmion‐Pair

**Figure 1:**
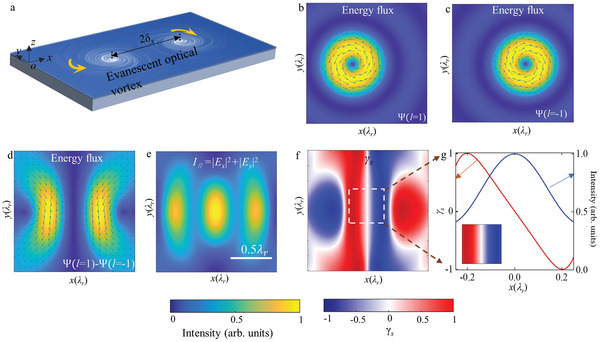
Illustration of the structured spin texture in a skyrmion‐pair. a) Schematic of the skyrmion‐pair that is composed of a pair of separated evanescent optical vortices with topological charges of opposite‐signs. b) Energy flux of an evanescent optical vortex with *l* = 1 that is left‐shifted by *δ_x_*. c) Same as (b) but with *l* = −1 and right‐shifted. d–f) Energy flux, in‐plane electric field intensity, and spin distribution, respectively, when the two evanescent optical vortices in (b) and (c) are superposed. The spacing between the two evanescent optical vortices is 2*δ_x_* = 0.36*λ_r_*. g) Spin variation (left vertical axis) and the intensity of the in‐plane electric field (right vertical axis) against the lateral position along the *x*‐axis for *y* = 0. Inset: a zoomed‐in image of the displacement‐sensing area within the dotted square in (f). The scale bar in (e) represents 0.5*λ_r_*, where *λ_r_* is the wavelength of the evanescent optical vortices; the areas in (b–e) are the same.

To explore the spin texture in the skyrmion‐pair, a pair of evanescent optical vortices of opposite topological charge in a source‐free, homogeneous, and isotropic medium (**Figure** **1**a) was considered to form a pair of close photonic skyrmions of opposite skyrmion numbers. The photonic skyrmions are located at (−*δ_x_*,0) and (*δ_x_*,0), with skyrmion numbers equal to +1 and −1, respectively. The Poynting vectors describing the energy flow of photons for each evanescent optical vortex (Figure 1b,c) prescribe a chiral swirl of spin vectors that define a single photonic skyrmion. The electromagnetic field properties of the skyrmion‐pair are expressible as a superposition of Hertz potentials for the two evanescent optical vortexes, $\Psi =\Psi_{1}+\Psi_{2}e^{i\varphi_{0}}$, in which *φ*_0_ denotes the initial phase delay between Ψ_1_ and Ψ_2_. In cylindrical coordinates, when the evanescent optical vortices are out of phase (*φ*_0_ = *π*), the total Hertz potential becomes

(1) $$\Psi =\Psi_{1}-\Psi_{2}=J_{1}\left(k_{r}r_{1}\right)e^{i\varphi_{1}}e^{-k_{z}z}-J_{-1}\left(k_{r}r_{2}\right)e^{-i\varphi_{2}}e^{-k_{z}z}$$

where *r_i_* and *φ_i_* denote, respectively, the distance and azimuthal angle of the observation point with respect to the center of the *i*‐th photonic skyrmion (*i* = 1,2), *k_r_* and *k_z_* the transverse and longitudinal wave‐vector components satisfying $k_{r}^{2}-k_{z}^{2}=k_{0}^{2}$ with *k*_0_ the free space wave‐vector, and *J*_1_ denotes the first‐order Bessel function of the first kind.

When the separation of the two photonic skyrmion is not comparable to the wavelength (*δ_x_* ≪*λ*_r_ = 2*π*/*k_r_*), strong coupling occurs between the vortex pair. The Hertz vector then approximates to $\Psi \approx Axe^{-i\eta k_{r}y}e^{-k_{z}z}$, where *A* and *η* are constants determined by *δ_x_* and *λ_r_* (see Supporting Information Note S1 for detail of the calculation). Subsequently, with the Hertz potential, the Poynting vector becomes^[^ ^31^ ^]^

(2) P∝−Im(Ψ∇Ψ∗)=−A2ηkrx2e−2kzzey

which reveals that *P_y_* dominates within the central region of the system and remains constant along the *y* direction (Figure 1d). The spin–orbit coupling in a transverse magnetic/electric polarized evanescent wave is governed by the *curl*‐relationship between the SAM and Poynting vector; explicitly, $S=\frac{1}{2\omega^{2}}\nabla \times P$, where *ω* denotes the angular frequency of the wave.^[^ ^24^ ^]^ In consequence, a linear variation in the out‐of‐plane component *S_z_* of the SAM with respect to the lateral position along the *x* direction is achieved with

(3) $$S_{z}=\frac{1}{2\omega^{2}}\frac{\partial P_{y}}{\partial x}\propto -2\eta k_{r}x$$

Remarkably, the intensity of the in‐plane electric field *I*_||_ = |*E_x_* |^2^ *+*|*E_y_* |^2^ (which is related to *S_z_*) reaches a maximum and is nearly constant in the central region where the SAM exhibits linearity (Figure 1e). This is immensely beneficial in detection applications and in improving the signal‐to‐noise ratio of the sensing configuration. To eliminate influences from the intensity of the field, a normalized SAM related to the local polarization is introduced; specifically, $\gamma_{s}=4\frac{\omega }{\varepsilon }\frac{S_{z}}{I_{//}}$, where *ε* denotes the permittivity of the medium. Here, *γ_s_* = +1(‐1) signifies pure right (left)‐handed circular polarization, *γ_s_* = 0 linear polarization, and fractional values represent elliptical polarization. By optimizing the distance between the pair of skyrmions, *δ_x_* = 0.18*λ_r_* (see Supporting Information Note S2 for the dependence of the spin distribution on spacing), the range in linearity in the *x*‐direction as well as the range in uniformity in the *y*‐direction for the spin state can be optimized (Figure 1f,g), making the skyrmion‐pair excellent for applications in ultrasensitive displacement sensing. It is worth to mention here that the pattern of the above spin structure is prescribed by a scalar wave function Ψ, without needing to deal with a complex vector and phase information normally associated with electromagnetic fields.

### 2.2 Excitation and Characterization of the Spin Texture

**Figure 2:**
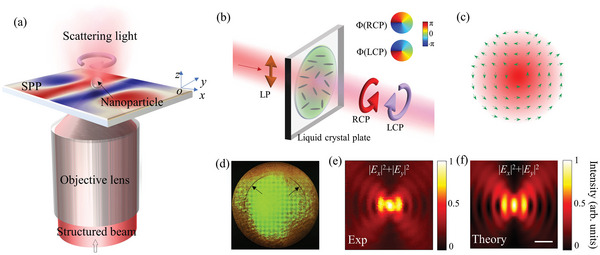
Experimental excitation and mapping of a skyrmion‐pair as an example of surface plasmon polaritons (SPPs). a) Schematic of the experimental configuration, for which the desired SPP field is formed at a silver surface and excited with an oil‐immersed TIRF lens. A polystyrene nanoparticle was employed to scatter the SPP field to the far field for analysis. b) Phase modulation on the incident linearly polarized beam with a liquid‐crystal plate to form a structured beam as used in (a). The liquid‐crystal plate is chiral dependent and codes the two circularly polarized components in a linearly polarized beam with opposite phases. c) Structured beam formed after the liquid‐crystal plate and subsequently focused onto the silver surface using an objective lens; the arrows indicate local polarization orientations. d) Image of the reflected beam captured at the back Fourier plane of the objective lens, where a pair of dark arcs appear confirming an SPP excitation at the silver surface. e) Mapped intensity distribution of the SPP obtained by raster scanning the polystyrene nanoparticle. f) Corresponding theoretical result using the Richard–Wolf theory; the scale bar represents 0.5 µm.

In experiments to confirm our spin‐based displacement‐sensing mechanism, we investigated surface plasmon polaritons (SPPs) supported at a dielectric–metal interface. ^[^ ^32^ ^]^ The configuration chosen for the sensing system (**Figure** **2**a, see Supporting Information Note S5: Materials and methods for the experimental setup) produces an SPP field excited on a silver surface under tight‐focusing conditions established by an oil‐immersed total internal reflection (TIRF) objective lens. To achieve lateral antishifts of the counter‐rotating SPP vortices, the incident linearly polarized Gaussian beam is coded with a specially designed phase imposed by a liquid‐crystal plate before being directed onto the TIRF lens (Figure 2b).

From the orientation of the liquid‐crystal molecules, the liquid‐crystal plate performs the phase modulation via a geometric phase. This modulation is expressed mathematically using the Jones‐matrix expression for the electric field ***E*** of the beam after the plate, specifically,

(4) $$E=\frac{1}{\sqrt{2}}\left[\left(\begin{array}{c} 1\\ -i \end{array}\right)e^{i\Phi }+\left(\begin{array}{c} 1\\ i \end{array}\right)e^{-i\Phi }\right]$$

where Φ denotes the phase term coded into the liquid‐crystal plate, which is designed to give Φ = 2 *πδ* cos*ϕ*, with *ϕ* the shifting angle with respect to the *x*‐axis and *δ* = *δ_x_*/*λ*_spp_ the shift‐fraction along the *x*‐direction relative to the SPP wavelength. This phase term Φ realizes a lateral shift of the SPP vortex without modifying its wavefront (see Supporting Information Note S3 for a Fourier analysis of the shift). Moreover, from Equation (4), this geometric phase is chiral dependent, implying an opposite phase for the left‐handed (LCP) and right‐handed circularly‐polarized (RCP) light. Because incident linear polarization may be considered to be a superposition of the above two circular polarizations, the two circularly polarized (CP) components are coded with conjugated phase terms by passing a linearly polarized beam through the liquid‐crystal plate (Figure 2b). Moreover, an SPP vortex can be excited by a CP beam under tight‐focusing conditions. On the silver surface, the two CP components excite a pair of SPP vortices of opposite topological charge, and their opposite phases from the liquid‐crystal plate induce a lateral splitting of the vortices to create the desired spin structure (Figure 1f).

The above process reassembles the polarization state of an incident beam forming a complex vector beam (Figure 2c, arrows indicate local polarization orientations). Only the radially polarized (*p*‐polarized) component excites the SPP when this structured vector beam is tightly focused onto the silver surface with the TIRF lens. Accordingly, the reflected beam captured at the back Fourier plane of the TIRF lens exhibits two dark arcs at the beam's cross‐sectional plane where *p*‐polarization is dominant (Figure 2d), thus validating the creation of an SPP excitation. Subsequently, the near‐field intensity distribution of the in‐plane electric field component of the excited SPP may be mapped by raster scanning a polystyrene nanoparticle over the SPP field. The mapped result (Figure 2e) agrees well with the theoretical result obtained using the Richard–Wolf vectorial diffraction theory (Figure 2f) (see Supporting Information Note S4 for calculation details).

**Figure 3:**
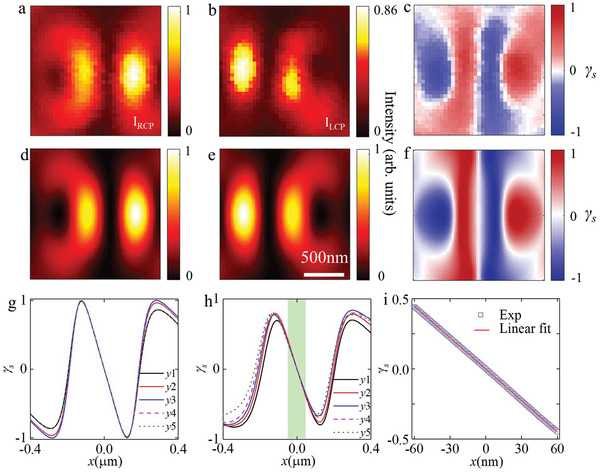
Characterizing the optical spin distribution of the structured surface plasmon polaritons (SPPs) field. a,b) Mapped intensity distributions of the RCP and LCP components of the transverse field component of SPP. c) Constructed spin distribution from (a) and (b) obtained from *γ _s_* = (*I*_RCP_ *‐I*_LCP_)/(*I*_RCP_ *+I*_LCP_). d–f) Corresponding theoretical results obtained from the Richard–Wolf theory; the scale bar in (e) represents 500 nm. g,h) Cross‐sectional plots at various *y*‐positions of results from calculations and experiments, respectively; *y*1–*y*5 denote the *y*‐positions from −100 to 100 nm at 50‐nm increments. i) A typical characteristic sensing curve from the central region (green area highlighted in (h) obtained with a scanning increment of 1 nm.

We extracted the two CP components (*I*_RCP_, *I*_LCP_) of the in‐plane electric field of the SPP, to construct the spin parameter using the expression, $\gamma_{s}=4\frac{\omega }{\varepsilon }\frac{S_{z}}{I_{//}}=\;\left(I_{\mathrm{RCP}}-I_{\mathrm{LCP}}\right)/\left(I_{\mathrm{RCP}}+I_{\mathrm{LCP}}\right)$.^[^ ^33^ ^]^ In the experiment, the extraction is achieved using a *λ*/4‐waveplate and a linear polarizer with their axes angled at ±45° for analyzing the scattering radiation from the nanoparticle. The mapped intensity distributions for the RCP and LCP components and the constructed spin distribution were shown in **Figure** **3**a–c, respectively. To eliminate the vibrational signal from the system, low‐pass filtering conducted in the Fourier domain was performed on the raw data from the experiment (see Supporting Information Note S6 for data‐processing details). The corresponding theoretical results, provided for comparison (Figure 3d–f), coincide well with the experimental results. The mapped results reveal the formation of the desired spin structure, thus enabling spin‐based displacement sensing.

The simulation and experimental plots of the cross‐sections at different *y*‐positions from −100 to 100 nm at 50 nm increments (Figure 3g,h) demonstrate the stability of the spin structure along the *y*‐direction. Theoretically, the *y*‐direction stability is well maintained within a range of about 200 nm along the *x*‐axis in the central region (Figure 3g), whereas in the experiment, this region is maintained at least to 120 nm (green area highlighted in Figure 3h). In a subsequent experiment, a typical magnified portion of the curve from the central region (Figure 3i) was obtained at scanning increments of 1 nm.

### 2.3 Application for Displacement Sensing

**Figure 4:**
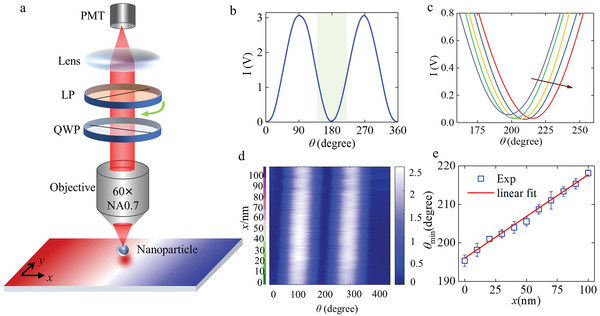
A proof‐of‐concept experiment to sense the movement of the polystyrene nanoparticle with a rotating linear polarizer. a) Schematic of the setup showing only the collection part; the excitation part is shown in Figure 2a. QWP: quarter wave plate, LP: linear polarizer, PMT: photomultiplier tube. b) Intensity‐varying curve when rotating the linear polarizer through a whole turn. The signal was captured with a photomultiplier tube PMT after the CP analyzer. c) Shift in intensity curves when moving the polystyrene nanoparticle with 20 nm step. d) Intensity distribution when the particle is moved 100 nm in 10 nm increments. The color label on the left of the figure represents where the particle is located. In the labeled area of the same color, the particle is remained and waiting for data acquisition ten times. e) Plot of the dip‐angle (i.e., angles corresponding to the dip intensities) against the lateral position of the particle.

A proof‐of‐concept experiment (**Figure** **4**a) was performed to validate the effectiveness of our approach and allows us to measure displacements by simply measuring the elliptical polarization state. An objective lens with NA = 0.7 was employed to collect the scattering radiation from the polystyrene nanoparticle, followed by a fixed *λ*/4‐waveplate and a rotating linear polarizer for analyzing the elliptical polarization state of the scattered radiation from the nanoparticle. The polarizer was mounted on a rotational stage (DDR05, Thorlabs) with a minimum incremental rotation of 0.00036°. A typical intensity‐varying curve (Figure 4b) when rotating the linear polarizer easily features a dip in intensity. Low‐pass FFT filtering was performed on the primary signals. When the particle was moved relative to the spin structure, a set of intensity curves near the dip region is obtained (Figure 4c); we then found the change in the dip‐angle *θ*_dip_ during the movement. Figure 4d shows the contour map of the intensity when the particle was moved over a distance of 100 nm at 10 nm increments. To diminish systematic error, the procedure at each position was repeated ten times, and the measured dip‐angles were averaged to give a final value. The fluctuation in the standard deviation at each position is caused by vibrations of the system but also arises from the limited number of sampling points obtained at each position. The dependence of the result on the lateral position along the *x*‐direction (Figure 4e) shows a change of 0.22° nm^−1^ in dip‐angle when moving the stage, which demonstrates a potential resolution of the displacement of about 1.7 pm (corresponding to the minimum incremental rotation of 0.00036° of the rotation stage). Moreover, the potential resolution of the displacement sensing system can be further improved by the improvement of the angular resolution of the rotation stage.

## 3 Conclusion

In summary, we proposed an approach of spin manipulation to study the spin texture with subwavelength features, which was achieved in a pair of close photonic Skyrmions of opposite Skyrmion numbers. By analyzing the Poynting vector and spin texture of the skyrmion‐pair, we demonstrate that the SAM varies linearly along the line connecting the centers of the two interacting skyrmions, while remaining almost constant in the transverse direction. Adjusting the space distance of the skyrmions in the skyrmion‐pair, the range in linearity is up to 0.4*λ*, at which the electric field intensity could be maximized. The optimized spin texture was created, detected, and then appropriately applied in the displacement‐ and position‐sensing system. The sensitivity of the spin‐based displacement sensing can reach pico‐meters over distances of at least 100 nm in the experiment. Because the photonic skyrmion is polarization‐independent, future studies may apply transverse electric‐polarized evanescent waves subject to the same mechanism to construct other spin textures and alternative displacement‐sensing techniques. Under such circumstances, the longitudinal electric field component is absent, and the detected transverse field intensity is stronger; hence, the stability in sensing can be further enhanced. The proposed spin‐based manipulation methods could provide novel insights in spin photonics and have potential applications in optical nanometrology, localization microscopy, and lithography mask alignment.

## Conflict of Interest

The authors declare no conflict of interest.

## Author Contributions

L.D. developed the concept and designed the experiment. A.Y., X.L., P.S., and L.D. carried out the analytical and numerical calculation. A.Y., F.M., and M.L. performed the experiments. L.D., A.Y., and X.L. wrote the manuscript. L.D. and X.Y. supervised the work. All the authors discussed the results and commented on the manuscript.

## Supporting information

Supporting Information

## Data Availability

The data that support the findings of this study are available in the Supporting Information of this article.
