# Supplementary material for: Spin‐Manipulated Photonic Skyrmion‐Pair for Pico‐Metric Displacement Sensing
Source: Adv Sci (Weinh). 2023 Feb 25;10(12):2205249. doi: 10.1002/advs.202205249 (PMC10131799; doi:10.1002/advs.202205249)
Supplement: Supplementary file 1 — Supporting Information [file ADVS-10-2205249-s001.pdf]

ADVANCED SCIENCE
Open Access

Supporting Information

for *Adv. Sci.*, DOI 10.1002/advs.202205249

Spin-Manipulated Photonic Skyrmion-Pair for Pico-Metric Displacement Sensing

Aiping Yang, Xinrui Lei, Peng Shi, Fanfei Meng, Min Lin, Luping Du and Xiaocong Yuan**

Supplementary Materials for

Spin-manipulated photonic skyrmion-pair for pico-metric displacement sensing

Aiping Yang, Xinrui Lei, Peng Shi, Fanfei Meng, Min Lin, Luping Du* and Xiaocong Yuan*

Nanophotonics Research Centre, Institute of Microscale Optoelectronics, Shenzhen University, Shenzhen, 518060, China

*Corresponding author. Email: *lpdu@szu.edu.cn* and *xcyuan@szu.edu.cn*.

Supplementary Note 1: Hertz potential calculation and approximation for the Skyrmion pair

The superposition of Hertz vector potentials for the evanescent optical vortices (Fig. 1A) is expressed as

$$\Psi = \Psi_1 - \Psi_2 = J_1(k_r r_1)e^{i\varphi_1}e^{-k_z z} - J_{-1}(k_r r_2)e^{-i\varphi_2}e^{-k_z z} = J_1(k_r r_1)e^{i\varphi_1}e^{-k_z z} + J_1(k_r r_2)e^{-i\varphi_2}e^{-k_z z}, \tag{S1}$$

where r_i, φ_i (i=1,2) denote the polar radius and angle with respect to the center of the i-th evanescent optical vortex. By introducing the relation $J_l(k_r r)i^l e^{il\varphi} = \frac{1}{2\pi}\int_0^{2\pi} e^{ik_r(x\cos\phi + y\sin\phi)}e^{il\phi}d\phi$, (with $r = \sqrt{x^2+y^2}$, φ=arg(x,y)) into equation (S1), the Hertz potential becomes

$$\begin{aligned}\Psi &= -\frac{i}{2\pi}\int_0^{2\pi}\{e^{ik_r[(x+\delta_x)\cos\phi + y\sin\phi]}e^{i\phi} + e^{ik_r[(x-\delta_x)\cos\phi + y\sin\phi]}e^{-i\phi}\}d\phi \\ &= -\frac{i}{\pi}\int_0^{2\pi}\{\cos(\phi + 2\pi\delta\cos\phi)e^{ik_r(x\cos\phi + y\sin\phi)}d\phi\end{aligned}, \tag{S2}$$

where $\delta = \frac{\delta_x}{\lambda_r}$. When δ_x is not comparable to λ_r, $\cos(\phi+2\pi\delta\cos\phi)$ can be approximated by applying the Taylor expansion for the sine and cosine functions near 0. After omitting the terms of high order, we find

$$\cos(\phi+2\pi\delta\cos\phi) \approx \cos\phi(1-\frac{3\pi^2\delta^2}{2}) - \frac{\pi^2\delta^2}{2}\cos 3\phi - \pi\delta\sin 2\phi(1-\frac{\pi^2\delta^2}{3}) + \frac{\pi^3\delta^3}{6}\sin 4\phi. \tag{S3}$$

Substituting equation (S3) into equation (S2) and omitting the high-order terms, the Hertz potential approximates to

$$\Psi \approx -2i[i(1-\frac{3\pi^2\delta^2}{2})J_1(k_r r)\cos\varphi + \pi\delta(1-\frac{\pi^2\delta^2}{3})J_2(k_r r)\sin 2\varphi]. \tag{S4}$$

Expanding the Bessel functions near 0, equation (S4) becomes

$$\Psi \approx Ax(1-i\eta k_r y) \approx Axe^{-i\eta k_r y}, \tag{S5}$$

where $A = (1-\frac{3\pi^2\delta^2}{2})k_r$ and $\eta = \frac{\pi\delta}{2}(\frac{1-\frac{\pi^2\delta^2}{3}}{1-\frac{3\pi^2\delta^2}{2}})$.

Supplementary Note 2: Spacing-dependent spin distributions of the Skyrmion pair

Figure S1 shows the spacing-dependent spin distributions of the Skyrmion pair involving opposite topological charges. From left to right, the shifting distance of the two superposed evanescent optical vortice are δ_x=0.06λ_r, 0.12λ_r, 0.18λ_r, 0.24λ_r, and 0.36λ_r. As can be seen, the spin distribution near the central region is optimized when δ_x=0.18λ_r for displacement sensing, giving rise to linearity along the x-axis and uniformity along the y-axis. Figure S2 shows the spin distributions of the skyrmion pair with opposite skyrmion numbers (+1 and −1) when distance of the cores is δ_x=0.18λ_r.

Supplementary Note 3: Fourier analysis of the shift of evanescent optical vortex by phase modulation

The out-of-plane electric field component of the evanescent optical vortex with unit topological charge can be expressed as $E_z \propto J_1(k_r r)e^{i\varphi}e^{-k_z z}$, which in the Fourier domain becomes

$$J_1(k_r\sqrt{x^2+y^2})\ e^{i\varphi} = \frac{i}{2\pi}\int_0^{2\pi} e^{-ik_r(x\cos\phi+y\sin\phi)}e^{i\phi}d\phi\,. \tag{S6}$$

After modulation with the phase term $\Phi = 2\pi\delta\cos(\phi-\theta)$, the electric field becomes

$$E_z \propto \frac{i}{2\pi}\int_0^{2\pi} e^{i2\pi\delta\cos(\phi-\theta)}\cdot e^{-ik_r(x\cos\phi+y\sin\phi)}e^{i\phi}d\phi\,. \tag{S7}$$

Replacing $2\pi\delta$ by $k_r\Delta r$, where $\Delta r=\sqrt{\Delta x^2+\Delta y^2}$ containing a $\Delta x=\Delta r\cos\theta$ shift along the x-direction and $\Delta y=\Delta r\sin\theta$ along the y-direction, equation (S7) reduces to

$$\begin{aligned}
&\frac{i}{2\pi}\int_0^{2\pi} e^{ik_r\Delta r\cos(\phi-\theta)}\cdot e^{-ik_r(x\cos\phi+y\sin\phi)}d\phi\\
&=\frac{i}{2\pi}\int_0^{2\pi} e^{ik_r(\Delta x\cos\phi+\Delta y\sin\phi)}\cdot e^{-ik_r(x\cos\phi+y\sin\phi)}e^{i\phi}d\phi\\
&=\frac{i}{2\pi}\int_0^{2\pi} e^{-ik_r[(x-\Delta x)\cos\phi+(y-\Delta y)\sin\phi]}e^{i\phi}d\phi\\
&=J_1(k_r\sqrt{(x-\Delta x)^2+(y-\Delta y)^2})e^{i\varphi_1}
\end{aligned} \tag{S8}$$

Comparing equations (S8) and (S6), we find that the phase term $2\pi\delta\cos(\phi-\theta)$ shifts the evanescent optical vortex without modifying its wavefront. In our work, we only consider displacements along the *x*-direction (θ=0); the phase term is then $2\pi\delta\cos\phi$.

Supplementary Note 4: Richard–Wolf method for calculation of the SPP field

The near-field distributions of SPP given in the manuscript and Supplementary Materials were calculated using the Richard–Wolf vectorial diffraction theory. On the surface of the silver film, the electric field is

$$E = A\int_0^{\alpha}\int_0^{2\pi}\psi(\theta_1,\phi)(\frac{k_2}{k_1})\underbrace{e^{ik_0z_0(n_2\cos\theta_2-n_1\cos\theta_1)}}_{term1}\underbrace{e^{i[k_2r\cos\theta_2+k_1r\sin\theta_1\cos(\phi-\phi_s)]}}_{term2}\sin\theta_1 d\theta_1 d\phi_1\,, \quad \text{(S9)}$$

where A is a constant, $\psi(\theta_1,\phi)=P(\theta_1,\phi)B(\theta_1,\phi)$ denotes the vector pupil distribution containing information of the polarization, phase, and amplitude of incident electric field, k_0 the wave-vector of incident light, k_1 and k_2 denote the wave-vectors before and after the focal plane, respectively, θ_1 and ϕ the tangential angle with respect to the *z*-axis and the azimuthal angle with respect to the *x*-axis, and n_1 and n_2 the refractive indexes of glass substrate and metal film. The additional *term1* describes the effects of the refractive index mismatch between the two media, and *term2* reflects the conversion from Cartesian to spherical coordinates.

In our calculation, given the electric field of incident light, equation (4) in the main text, the electric field distribution is then

$$E_{n2} = A\int_0^{\alpha}\int_0^{2\pi}\sqrt{\cos\theta_1}\,\frac{k_2}{k_1}e^{ik_0z_0(n_2\cos\theta_2-n_1\cos\theta_1)}e^{i[zk_2\cos\theta_2+k_1r\sin\theta_1\cos(\varphi-\varphi_s)]}F(t_p,t_s)E\sin\theta_1 d\theta_1 d\varphi\,, \quad \text{(S10)}$$

where $F(t_p,t_s)$ denotes a function of the Fresnel coefficients.

Supplementary Note 5: Materials and methods for the experimental setup

Our experimental setup (Fig. S3) involves a laser beam of wavelength 632.8 nm being modulated by a liquid crystal plate to generate a structured beam. Prior, a half wave plate (HWP) was used to

tune the polarization of the beam suitable for the liquid-crystal plate. Then, using a telescope, the structured beam was tightly-focused with a TIRF lens (Olympus, 100×, NA=1.49) onto a sample which was fixed on a piezo-stage (Physik Instrumente, P-545). To observe the excitation of the SPPs, a CCD camera was placed at the back focal plane of the TIRF lens to capture the reflected beam. The sample consists of a 50-nm-thick silver film deposited on a silica coverslip, with a polystyrene nanoparticle attached on the silver surface. The polystyrene nanoparticle was employed as a near-field probe to scatter out the structured evanescent field. The size of the nanoparticle was carefully selected (320 nm in this work) to sense only the transverse field of the SPPs. From the symmetry of the particle, the far-field scattering radiation has an elliptical polarization that is the same as the local polarization state of the evanescent field right below the nanosphere and therefore is able to characterize the spin information of the system.

The scattered light from the polystyrene nanoparticle that contains the spin information of SPP was collected by another objective lens (Olympus, 60×, NA=0.7). A beam splitter then split the light into two paths, with one directed toward a CCD camera to image the polystyrene nanoparticle and the other toward a polarization analyzing system comprising a $\lambda/4$ waveplate and a linear polarizer. The polarizer was mounted on a rotational stage (DDR05, Thorlabs) with a minimum incremental rotation of 0.00036°. To reduce the impact from direct transmitted light, a circular opaque mask designed specifically for this purpose was inserted before the TIRF lens. Furthermore, a collection fiber of a core diameter 50μm was used as a pinhole to direct the scattered light into a photo-multiplier tube (PMT, Hamamatsu). After the optical signal was converted to an electrical signal through the PMT, a 40 Hz–60 Hz band stop filter was employed to reduce background noise. The filtered signal was recorded using a data acquisition card (DAQ).

Sample preparation

A silver film of thickness 50nm was deposited onto a clean glass coverslip (thickness: 175 μm) by electron beam evaporation. This coated substrate was then immersed in a 16-Amino-1-hexadecanethiol hydrochloride solution for ~15min to form a self-assembled monolayer (SMA) on the surface of the film to form a SMA-Ag substrate. After the substrate was taken out, it was rinsed using deionized (DI) water and immediately dried using a nitrogen stream. A droplet of adequate diluted polystyrene nanoparticle suspension was dropped onto the prepared SAM-Ag substrate and allowed to evaporate naturally. Finally, the sample was again rinsed with DI water and dried using a nitrogen stream before using.

Supplementary Note 6: Details of data acquisition

During information acquisition, the stability of the system is affected by the vibration of the optical system and current noise of the equipment. The vibration of the optical system, including the optical platform and experiment setup, is a low-frequency signal of about 50Hz in our experiment. As a result, a band-stop filter with a spectrum width of 40Hz–60Hz was placed before the DAQ (Fig. S3). In contrast, the current noise generated by dark current from the PMT and DAQ is a high-frequency signal. During data processing, low-pass FFT filtering was performed on the original data curves using the algorithm. Fig. S4(a)-(d) presents the results of the data acquisition process that yielded figure 3h in the main-text. The FFT calculation was performed on the detection signals (left-hand and right-handed intensity signals obtained in the experiment [Fig. S4(a)]. Then the low frequency part [Fig. S4(b), green area] was retained and the signals in the Fourier domain was shown in Fig. S4(c). After low-pass FFT filtering, the final signal [Fig. S4(d)] was obtained.

Supplementary figures

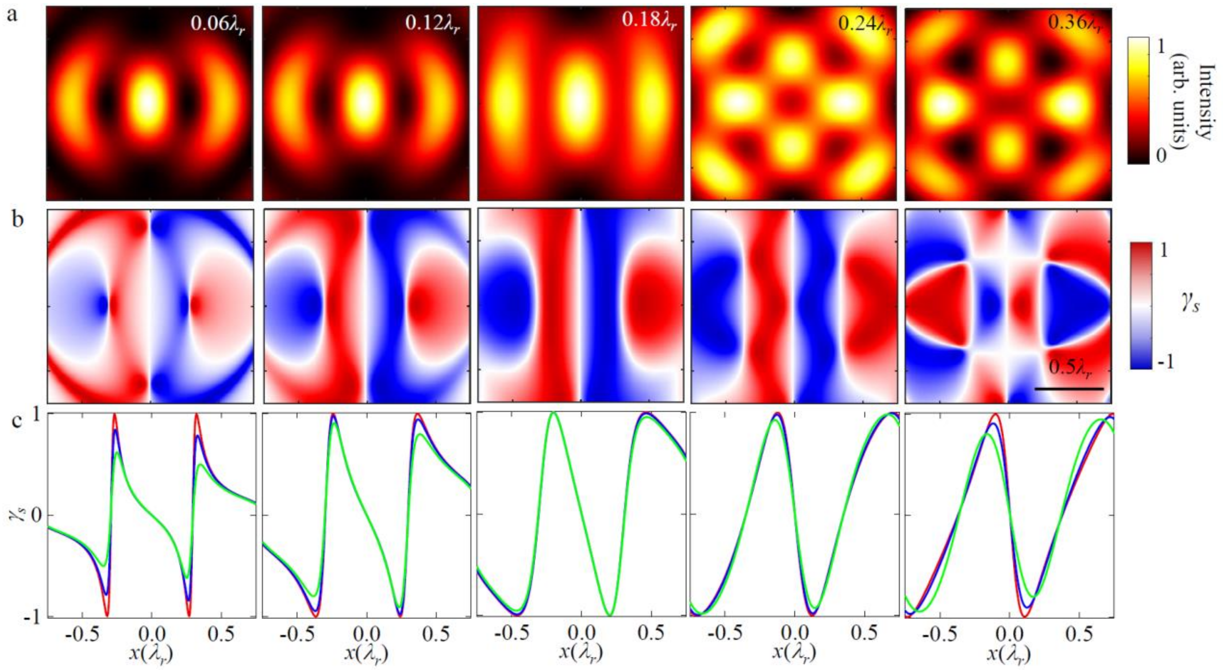

Fig. S1. **Spacing-dependent spin distributions of the skyrmion pair with opposite skyrmion numbers (+1 and −1).** From left to right, the distance of the cores is δ_x=0.06λ_r, 0.12λ_r, 0.18λ_r, 0.24λ_r, and 0.36λ_r, respectively. (a) Intensity distributions of the transverse field component. (b) Corresponding spin distributions γ_s, the areas in (a) and (b) are the same. (c) Cross-sectional plots at various y-positions. The plots for y=0, y=0.05λ_r, and y=0.1λ_r are marked in red, blue, and green, respectively.

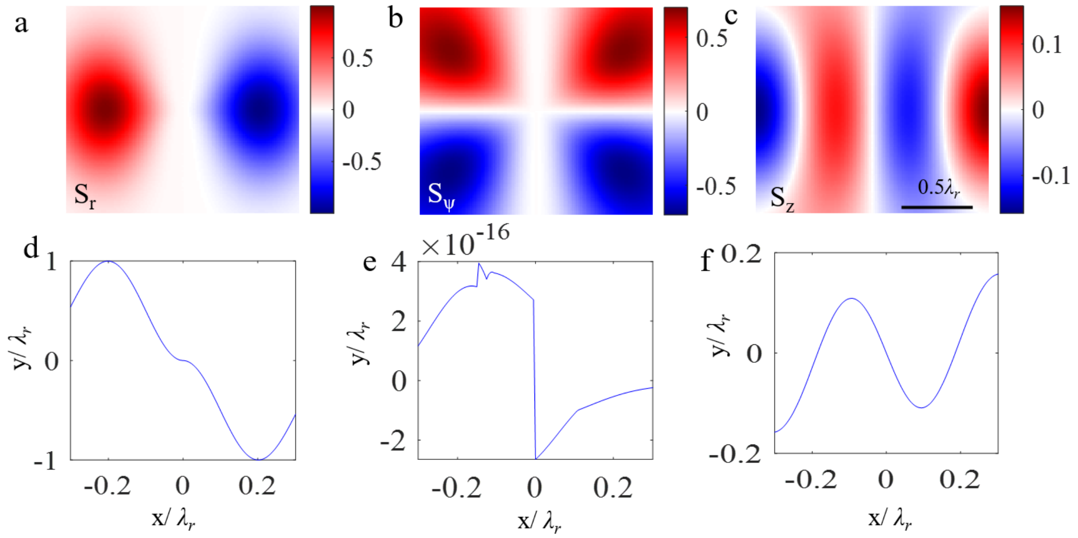

Fig. S2. **Spin distributions of the skyrmion pair with opposite skyrmion numbers (+1 and −1) when distance of the cores is $\boldsymbol{\delta_x = 0.18\lambda_r}$.** (a-c) The spin distribution S_r, S_ψ, S_z, respectively, and (d-e) the cross-line along *x*-axis.

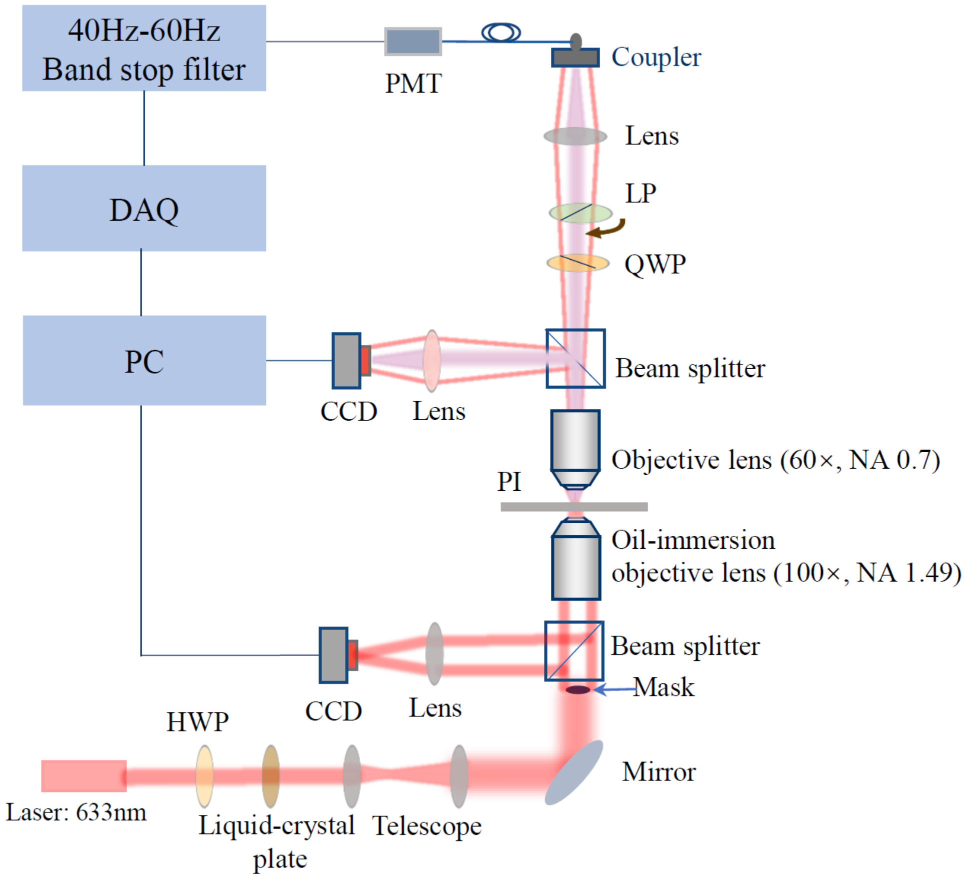

Fig. S3. **Experimental setup for the spin-based optical sub-nanometer metrology.** HWP: half wave plate, QWP: quarter wave plate, LP: linear polarizer, PMT: photo-multiplier tube, DAQ: data acquisition card, PC: personal computer. The incident wavelength is 633 nm.

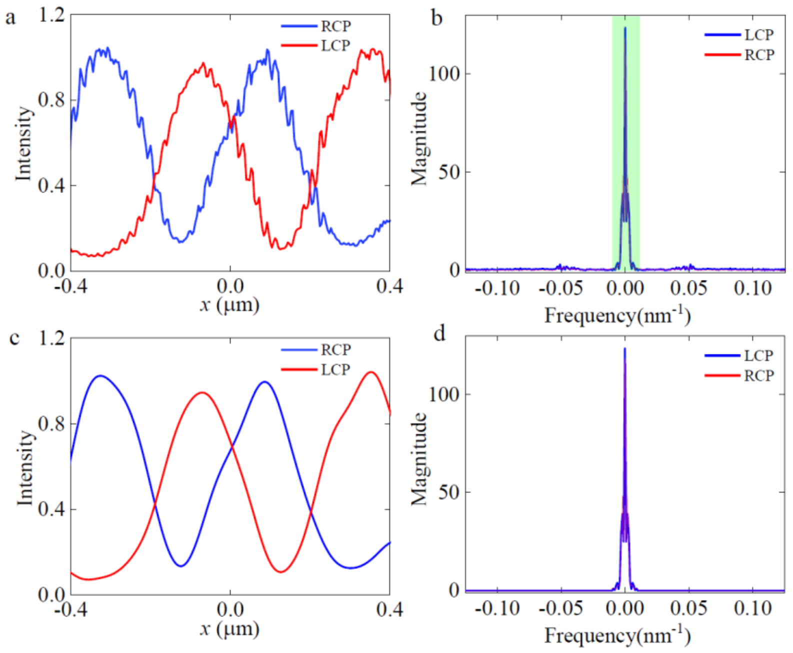

Fig. S4. **FFT low pass filtering for the experimental signal.** (a) Original signal obtained in the experiment. (b) Signal in the Fourier domain obtained using the FFT algorithm; the low-frequency part highlight in green was retained. (c)Retained signals in the Fourier domain. (d) Signal after low-pass FFT filtering.
